# The main WAP isoform usually found in camel milk arises from the usage of an improbable intron cryptic splice site in the precursor to mRNA in which a GC-AG intron occurs

**DOI:** 10.1186/s12863-018-0704-x

**Published:** 2019-01-29

**Authors:** Alma Ryskaliyeva, Céline Henry, Guy Miranda, Bernard Faye, Gaukhar Konuspayeva, Patrice Martin

**Affiliations:** 1 INRA, UMR GABI, AgroParisTech, Université Paris-Saclay, 78350 Jouy-en-Josas, France; 2 Plateforme d’Analyse Protéomique Paris Sud-Ouest (PAPPSO), INRA, MICALIS Institute, Université Paris-Saclay, 78350 Jouy-en-Josas, France; 3 CIRAD, UMR SELMET, 34398 Montpellier Cedex 5, France; 4 Biological Technology Department, Al-Farabi Kazakh National University, Almaty, Kazakhstan

**Keywords:** Camel, Milk, Whey protein, Splicing, Genetic polymorphism

## Abstract

**Background:**

Whey acidic protein (WAP) is a major protein identified in the milk of several mammalian species with cysteine-rich domains known as four-disulfide cores (4-DSC). The organization of the eutherian WAP genes is highly conserved through evolution. It has been proposed that WAP could play an important role in regulating the proliferation of mammary epithelial cells. A bacteriostatic activity was also reported. Conversely to the other mammalian species expressing WAP in their milk, camel WAP contains 4 additional amino acid residues at the beginning of the second 4-DSC domain, introducing a phosphorylation site. The aim of this study was to elucidate the origin of this specificity, which possibly impacts its physiological functions.

**Results:**

Using LC-ESI-MS, we identified in *Camelus bactrianus* from Kazakhstan a phosphorylated whey protein, exhibiting a molecular mass (12,596 Da), 32 Da higher than the original WAP (12,564 Da) and co-eluting with WAP. cDNA sequencing revealed a transition G/A, which modifies an amino acid residue of the mature protein (V12 M), accounting for the mass difference observed between WAP genetic variants. We also report the existence of two splicing variants of camel WAP precursors to mRNA, arising from an alternative usage of the canonical splice site recognized as such in the other mammalian species. However, the major camel WAP isoform results from the usage of an unlikely intron cryptic splice site, extending camel exon 3 upstream by 12-nucleotides encoding the 4 additional amino acid residues (VSSP) in which a potentially phosphorylable Serine residue occurs. Combining protein and cDNA sequences with genome data available (NCBI database), we report another feature of the camel WAP gene which displays a very rare GC-AG type intron. This result was confirmed by sequencing a genomic DNA fragment encompassing exon 3 to exon 4, suggesting for the GC donor site a compensatory effect in terms of consensus at the acceptor exon position.

**Conclusions:**

Combining proteomic and molecular biology approaches we report: the characterization of a new genetic variant of camel WAP, the usage of an unlikely intron cryptic splice site, and the occurrence of an extremely rare GC-AG type of intron.

## Background

Camel milk is characterized by a high content of vitamin C (average content ranging between 50 and 250 mg/L), and endowed with a unique composition of protein components [1–3]. Its protein content (35–50 g/L) is rather high [4], with ca. 80% are caseins and 20% whey proteins that are soluble at pH 4.6 whereas caseins precipitate close to this pH. The casein fraction comprises 4 caseins (α_s1_-, α_s2_-, β- and κ-casein) encoded by four autosomal genes (*CSN1S1, CSN1S2, CSN2* and *CSN3*, respectively) mapped on chromosome 6 in cattle and goat [5, 6]. This fraction is rather complex with many splicing variants and post-translational modifications [3]. Whey proteins of camel milk mainly consist of α-lactalbumin (α-LA), glycosylation-dependent cell adhesion molecule 1 (GlyCAM1) or lactophorin which is closely related to the bovine proteose peptone component 3 (PP3), the innate immunity Peptido Glycan Recognition Protein (PGRP) and the Whey Acidic Protein (WAP).

WAP is a major whey protein identified in the milk of several species from eutherians as well as marsupial and monotremes [7]. It was first shown to be secreted in rodent milks [8], and a whey protein, rich in half-cystine residues (*n* = 16), showing strong similarities with rodents WAPs was characterized two years later in camel milk [9]. Then, the WAP has been identified in rabbits [10], porcine [11], wallaby [12], brushtail possum [13] and more recently in canine [14] milks. Camel WAP reaches an average concentration (157 mg/L) 10-folds lower than that (1500 mg/L) in rodents milk [15], whereas it is a hundred times higher (15 g/L) in rabbit milk [16]. Whey acidic protein (WAP) is expressed in the mammary gland under an extracellular matrix and lactogenic hormones regulation [17]. WAP gene expression is induced by prolactin, inhibited by progesterone, and strongly amplified by glucocorticoids [18].

**Fig. 1:**
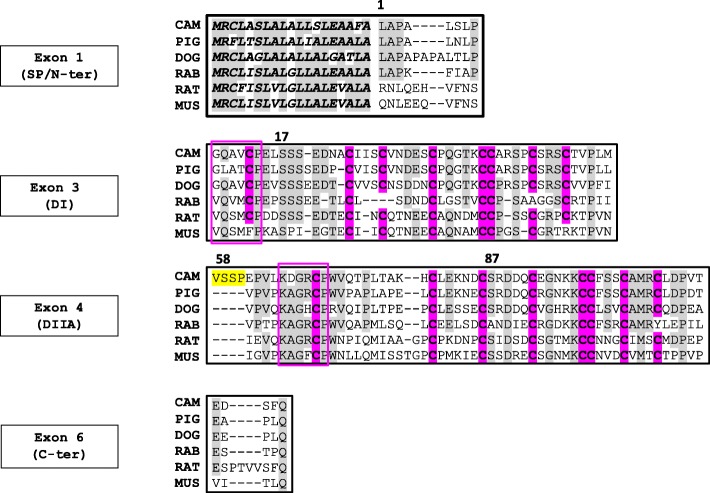
Multiple sequences alignment of WAP among Eutherian species including camel (NCBI, LOC105095719), pig (O46655), dog (GenBank AAEX02035361, positions 25,184-23,606), rabbit (P09412), rat (G3 V718), and mouse (Q7M748). Four exons: E1, E3, E4 and E6 (numbering of the putative ancestral gene, proposed by [7], are given in black boxes. Exons 3 and 4 represent 4-DSC domains (DI and DIIA), while exons 1 and 6 indicate the signal peptide (SP) with the N-terminal part (N-ter) of the mature protein and the C-terminal part (C-ter) of the protein, respectively. WAP motifs are boxed in pink. Conserved Cysteine residues (C) in each 4-DSC domain are pink shaded. Residues identical in more than 3 animal species are grey shaded. Gaps are introduced to maximize similarities. Tetrapeptide VSSP, that is specific to camel WAP, is highlighted in yellow

The overall organization of the eutherian WAP genes is highly conserved through evolution [7, 19]. It is composed of 4 exons: E1, E3, E4 and E6 (Fig. 1). While the size of each exon remains rather conserved between species, intron size varies considerably. The first exon encodes the 5’-UTR, N-terminal signal peptide of 19 aa residues, and the first 8–10 aa residues of mature eutherian proteins. Exons 3 and 4 encode two cysteine-rich domains (DI and DIIA) known as four-disulfide cores (4-DSC) in eutherian species [8]. A third domain (DIII) encoded by exon 2 (missing in eutherian genes) is found in Monotrema and Marsupial species [7]. Exon 6 encodes the last 5–9 aa residues and the 3’-UTR while exon 5 (DIIB) is only used in Platypus and Marsupial species [7]. Even though, the promoter region of WAP gene is similar to house-keeping genes with weak or absent TATA signal [15], WAP is not found in all eutherian milks. The functionality of the gene encoding WAP has been lost in ruminants and primates due to a frameshift mutation [20]. Consequently, there is no WAP in the milk of ruminants and primates.

The presence of 4-DSC domains in cysteine-rich proteins led to their classification as the WAP gene family. Proteins containing WAP domains with a characteristic 4-DSC occur not only in mammals but also in birds, reptiles, amphibians and fish [21]. Each domain comprises eight cysteine residues with a core of six spatially conserved, while the remaining two are positioned at variable distances amino terminal from the core [22].

The sequence conservation of 4-DSC motifs across species is significant, and it seems likely that the region may be involved in the biological function of the molecule. WAPs share structural similarity with serine protease inhibitors containing WAP motif domains characterized by a four-disulfide core (4-DSC) [8]. Possible physiological functions of WAP have been proposed, based on its similarity to protease inhibitor [16]. Using in vitro and in vivo systems, Nukumi et al. [23] suggested that WAP plays an important role in regulating the proliferation of mammary epithelial cells by preventing elastase-type serine proteases from carrying out extracellular matrix laminin degradation. In addition, the same authors report a bacteriostatic activity of rat WAP against *Staphylococcus aureus* [24]. In marsupial, Sharp et al. [7] suggest that WAP may play also a role in the development of the young. WFDC2, a second WAP-like protein, is differentially expressed in the mammary gland of the tammar wallaby and provides immune protection to the mammary gland and the developing pouch young [25].

The present study was undertaken first to search for WAP genetic polymorphism in camel species (*Camelus bactrianus* and *Camelus dromedarius*) from Kazakhstan. The alignment of sequences of WAP from 5 Eutherian species in which the WAP gene is expressed reveals the occurrence of an additional sequence of 4 amino acid residues in the camel WAP (Fig. 1). We show that this insertion is due to the usage of an intron cryptic splice site. Finally, results reported here clarify discrepancies and erroneous data found in sequence databases (predicted sequence from genomic data) and literature. We also report that in camel, the gene encoding WAP comprises a rare GC-AG intron-type that represents less than 1% of annotated donor sites, which is at the origin of predicted sequence anomalies.

## Methods

### Milk sample collection and preparation

Raw milk samples were collected during morning milking on healthy dairy camels (*n* = 179) that belonged to two species: *C. bactrianus* (*n* = 72) and *C. dromedarius* (*n* = 65), and their hybrids (*n* = 42) at different lactation stages, ranging between 30 and 90 days postpartum. Those milk samples were skimmed as previously described [3] and skimmed milks and fat were stored at − 20 °C and − 80 °C, respectively, until analysis.

### Selection of Milk samples for analysis

A set of 58 milk samples, including individuals from each camel species and grazing regions, was selected, based on lactation stages and number of parities (from 2 to 14), for SDS-PAGE analysis. Then, eight (*C. bactrianus*, *n* = 3, *C. dromedarius*, n = 3, and hybrids, *n* = 2) of those 58 milk samples from three different regions exhibiting the most representative profiles were analyzed by LC-MS/MS (LTQ-Orbitrap Discovery, Thermo Fisher) after tryptic digestion of excised gel bands. Additionally, 30 milk samples (*C. bactrianus*, *n* = 10; *C. dromedarius*, n = 10; hybrids, n = 10), taken from the 58 milks analyzed by SDS-PAGE, were analyzed by LC-ESI-MS (Bruker Daltonics). One camel milk sample (*C. bactrianus*) displaying a WAP genetic polymorphism in LC-ESI-MS was selected for amplification of WAP cDNA by RT-PCR and cDNA sequencing.

### Milk fat globule collection - RNA extraction and single-Strand cDNA synthesis

Total RNA was extracted from milk fat globules (MFG) fraction stored at − 80 °C using LS Trizol (Invitrogen) following the protocol from the manufacturer, as described by Brenaut et al. [26]*.* Then, first-strand cDNA was synthesized as described [3]. One microliter of 2 U/μL RNase H (Invitrogen Life Technologies) was then added to remove RNA from heteroduplexes. Single-strand cDNA thus obtained was stored at − 20 °C.

### Genomic DNA isolation

Genomic DNA (gDNA) was isolated from fresh blood of *C. dromedarius* collected in EDTA using the Wizard® Genomic DNA Purification Kit (Promega Corporation, Madison, USA). Briefly, for 3 mL blood sample volume, 9 mL of Cell Lysis Solution was added and centrifuged at 2000 x *g* for 10 min at room temperature (RT), after incubating the mixture for 10 min, at RT. The supernatant was removed and, 3 mL of Nuclei Lysis Solution was added to the resuspended white pellet containing white blood cells. The solution was pipeted 5-6 times to lyse the white blood cells. Then, 1 mL of Protein Precipitation Solution was added to the nuclear lysate, and centrifuged at 2000 x *g* for 10 min, at RT. The supernatant was transferred to a 15 mL tube containing 3 mL isopropanol and centrifuged at 2000 x *g* for 1 min, at RT. Gently mix the solution and centrifuged at 2000 x *g* for 1 min, at RT. After decanting the supernatant, one sample volume of 70% ethanol was added to the DNA. After 1 min centrifugation at 2000 x *g*, the ethanol was aspirated using a drawn Pasteur pipette and the pellet was air-dried for 10–15 min. DNA was rehydrated by adding 250 μL of DNA Rehydration Solution and incubated at 65 °C for 1 h and stored at 4 °C.

### PCR amplification and DNA sequencing

cDNA and gDNA samples were amplified using primer pairs, of which sequences are given in Table 1, designed starting from the published *Camelus* gene sequence (NCBI, LOC105095719) and synthesized by Eurofins genomics (Ebersberg, Germany). PCR was performed in an automated thermocycler GeneAmp® PCR System 2400 (Perkin-Elmer, Norwalk, USA) with GoTaq® G2 Flexi DNA Polymerase Kit (Promega Corporation, USA). Reactions were carried out in 0.2 mL thin-walled PCR tubes, as described by Ryskaliyeva et al. [3], using the following PCR cycling conditions: denaturation of cDNA template at 94 °C for 2 min, 35 cycles at 94 °C for 45 s (denaturation), 58 °C for 30 s (annealing) and 72 °C for 1 min (extension), with a final extension step of 5 min at 72 °C. Sequencing of PCR fragments was performed using PCR primers from both strands, according to the Sanger method by Eurofins MWG GmbH (Ebersberg, Germany).

**Table 1:** Primers used to amplify the cDNA and gDNA target of the WAP gene

	Position	Primer	Sequence 5′- > 3′	nt^a^	Amplicon sizes	Tm, ^o^C
cDNA	5’-UTR	Forward	ATCTGTCACCTGCCTGCCACCTG	23	557	66
	3’-UTR	Reverse	TGAAGCTGAGTGGGTTTTTATTAGC	25		60
gDNA	intron 2	Forward	CAGCTGAGGCTGGCCCGCCTC	21	561	70
	intron 3	Reverse	GCTAGTCTGACACCCTCTCTCTA	23		62

^a^ *nucleotides*

### 1D sodium dodecyl sulfate polyacrylamide gel electrophoresis (SDS-PAGE) and protein identification by LC-MS/MS analysis

Both major and low-abundance proteins resolved by SDS-PAGE were identified by tandem mass analysis (LC-MS/MS) after excision of the relevant bands from the gel and trypsin digestion [28]. The SDS-PAGE conditions was that from Laemmli [3, 27].

### LC-ESI-MS

Fractionation of camel milk proteins and determination of their molecular masses were performed by coupling RP-HPLC to ESI-MS (micrOTOF™ II focus ESI-TOF mass spectrometer; Bruker Daltonics) as described [3]. Clarified milk samples (25 μL) were directly injected onto a Biodiscovery C5 reverse phase column (300 Å pore size, 3 μm, 150 × 2.1 mm; Supelco, France). Eluted proteins were detected by UV-absorbance at 214 nm and the effluent directly introduced to the mass spectrometer. Positive ion mode was used, and mass scans were acquired over a mass-to-charge ratio (m/z) ranging between 600 and 3000 Da [3].

## Results

### Nucleotide sequence of camel WAP cDNA

**Fig. 2:**
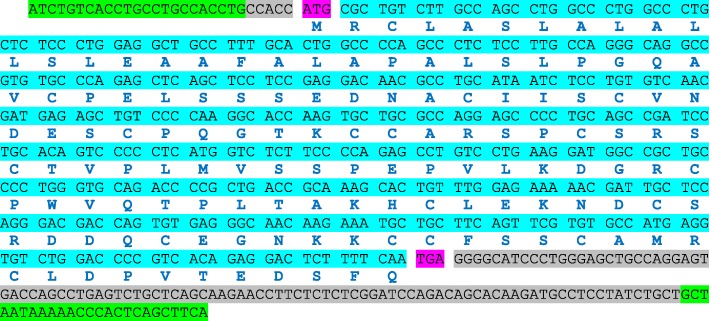
Nucleotide sequence of camel WAP cDNA. Primer pairs used for PCR and cDNA sequencing are highlighted in green. Start and stop codons are highlighted in fuchsia. AA residues encoded by triplet codons are bolded and in blue. The coding sequence and non-coding sequences are highlighted in cyan and grey, respectively

Using the rabbit and rodents WAP encoding gene sequences as references, we searched for the expected fourth exon of the WAP gene in the camel genome sequence (NCBI, LOC105095719). A pair of primers (Table 1) was thus designed, in the first exon upstream the coding sequence (forward) and overlapping the downstream putative AATAAA polyA signal (reverse), to amplify a cDNA fragment that was subsequently sequenced. The cDNA sequence of camel WAP thus obtained and given at Fig. 2, consists of 563 nucleotides encoding a 136 aa polypeptide of M_*r*_ 14,510.72 Da, including the signal peptide. The molecular mass of the corresponding mature protein is: 12,564.32 Da.

### Identification and characterization of a new WAP genetic variant in Bactrian camel milk

**Fig. 3:**
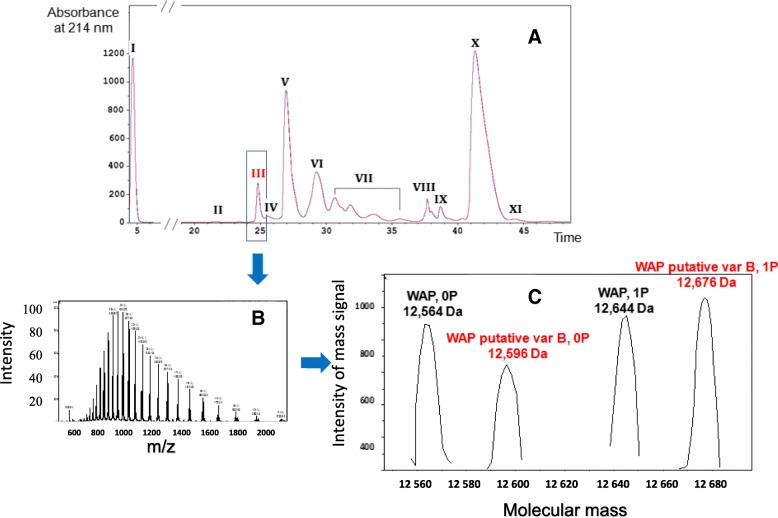
Milk protein profiling by LC-ESI-MS of a Bactrian camel milk from the Shymkent region. Eleven major milk protein fractions were identified from RP-HPLC profile (**3.A**) in the following order: glycosylated κ-CN A and B (I), non-glycosylated κ-CN A and B (II), WAP (III), shorter (∆ex16 and 13′) + short (∆ex16) isoforms of α_s1_-CN A and C (IV and V), α-LAC + α_s1_-CN A and C + (VI), α_s2_-CN* (VII), PGRP + α_s2_-CN* (VIII), LPO/CSA (IX), β-CN A and B (X) and γ_2_-CN A and B (XI). Multicharged-ions spectrum from compounds contained in fraction III (**3.B**). After deconvolution (**3.C**) the spectrum shows the presence of cognate camel WAP A-0P (12,546 Da) and 1P (12,644 Da) indicated in black, and molecular masses corresponding to a new WAP variant (named B) without (12,596 Da) and with (12,676 Da) one phosphate group, indicated in red. *Splicing variants of αs2-CN with different phosphorylation levels (Ryskaliyeva et al., submitted)

LC-ESI-MS analysis of a Bactrian camel milk from the Shymkent region (Fig. 3), revealed the presence, in peak III (Table 2), of two molecular masses differing by 80 Da (one phosphate group) 12,596 Da and 12,676 Da, besides the cognate WAP (12,564 Da and 12, 644 Da). Such a result and the small mass difference (32 Da) strongly suggested the existence of a novel WAP genetic variant, which had not been described so far in camels.

**Table 2:** Identification of WAP from molecular mass determination using LC-ESI-MS of a clarified Bactrian milk

Peak	Ret.Time, (min)	Observed M_*r*_ (Da)	Theoretical M_*r*_ (Da)	Protein description	UniProt accession	Intensity
III	24.31	12,564	12,564	WAP variant A, 0P	P09837	896
		12,596	n/a	WAP variant B, 0P	n/a	652
		12,644		WAP variant A, 1P		951
		12,677		WAP variant B, 1P		1059

*n/a* not applicable

**Fig. 4:**
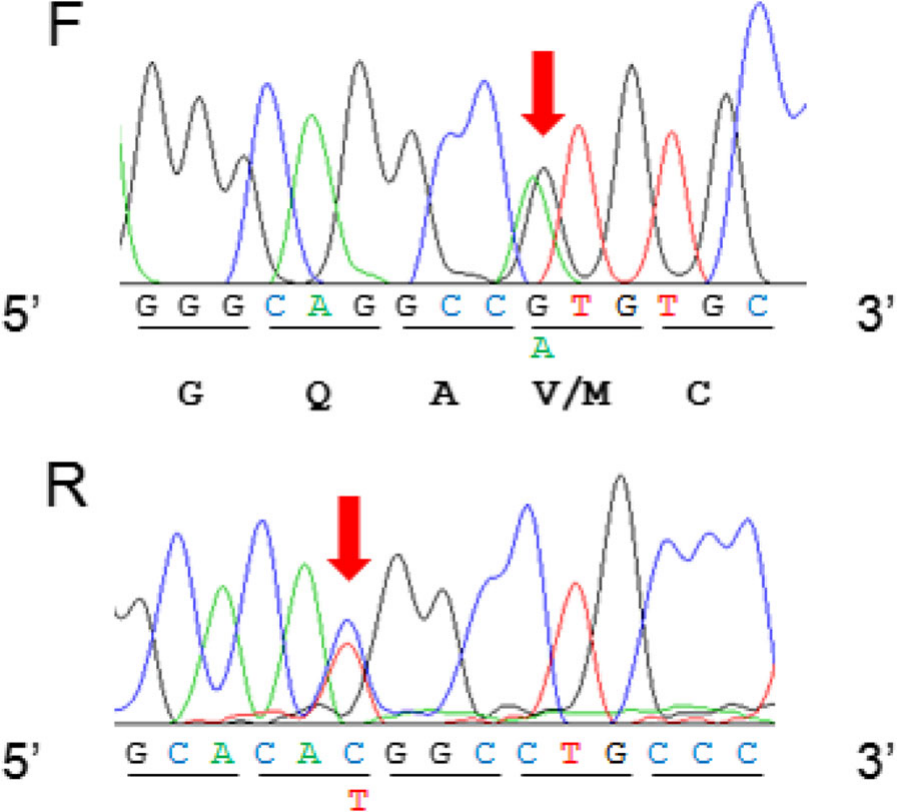
Partial sequence of WAP cDNA from *C. bactrianus* from Shymkent region. Nucleotides arising by manual reading, as G/A and C/T transitions, in forward (F) and reverse (R) sequences, respectively, are pointed out by vertical red arrows Nucleotides are clustered by three (codon) corresponding to aa residues (below codons in the forward sequence)

Nucleotide sequences of two unrelated individuals (including the Bactrian camel from the Shymkent region) were compared across the complete coding sequence of the camel WAP cDNA, in both directions. PCR yielded a fragment of the expected length (ca. 560 bp) for a complete mRNA open reading frame of 408 bp, demonstrating that the primary transcript was correctly spliced. However, examining the nucleotide sequence manually, a transition G/A may be easily noticed (Fig. 4), leading to the fourth codon change (GTG/ATG) of exon 2, confirmed by the reverse complement sequence. This single base substitution corresponds to a V/M amino acid substitution in position 12 of the mature protein, in agreement with the mass difference of 32 Da (V12 M, 99 Da = > 131 Da), found between WAP variants detected in LC-ESI-MS. We propose to name the camel WAP (V12) described by Beg et al. [9] as variant A and the newly identified variant (M12) as variant B. Consequently, molecular masses observed by LC-ESI-MS (12,596 Da, 12,676 Da) precisely correspond to unphosphorylated and phosphorylated (1P) isoforms of WAP variant B, respectively. This B variant which was found in only one (Bactrian) of the 30 camel milk samples analyzed in LC-ESI-MS, at the heterozygous state, appeared therefore to be rare in the Kazakh population. As far as variant A is concerned, the unphosphorylated isoform seems to be prevalent, with relative proportions between unphosphorylated and phosphorylated protein ranging between 70/30 and 55/45, whereas the phosphorylated isoform is predominant (40/60) with the Bactrian camel heterozygous A/B.

### Camel WAP may exist as two isoforms differing in size

LC-MS/MS implemented to confirm the identification of major camel milk proteins [3] revealed the existence of two WAP isoforms. Indeed, two tryptic peptides: SCTVPLM**VSSP**EPVLK and SCTVPLMEPVLK identifying camel WAP and differentiating in the presence or absence of the VSSP tetrapeptide (Table 3) were found in four of the eight individuals analyzed, belonging to the three camel species. However, the amount of the isoform lacking the tetrapeptide VSSP appeared too weak (1 spectrum vs 10 spectra with VSSP) to be detected by LC-ESI-MS that is less sensitive than LC-MS/MS.

**Table 3:** Sequences of WAP tryptic peptides identified by LC-MS/MS in the milk of a *C. bactrianus*

ID	UniProt accession	Species	Peptide sequence	aa residue		M*r*	Spectra
				Start	Stop		
1	P09837	*C. dromedarius*	LAPALSLPGQAVCPELSSSEDNACIISCVNDESCPQGTK	1	39	4174.90	1
2	P09837	*C. dromedarius*	LSLPGQAVCPELSSSEDNACIISCVNDESCPQGTK	5	39	3822.69	1
3	P09837	*C. dromedarius*	IISCVNDESCPQGTK	25	39	1707.77	1
4	P09837	*C. dromedarius*	VNDESCPQGTK	29	39	1234.54	2
5	P09837	*C. dromedarius*	**SCTVPLM** **VSSP** **EPVLK**	49	64	1743.90	6
6	P09837	*C. dromedarius*	**SCTVPLM** **VSSP** **EPVLKDGR**	49	67	2072.05	3
7	P09837	*C. dromedarius*	**PLM** **VSSP** **EPVLKDGR**	53	67	1624.87	1
8	P09837	*C. dromedarius*	DGRCPWVQTPLTAK	65	78	1628.82	1
9	P09837	*C. dromedarius*	CPWVQTPLTAK	68	78	1300.67	11
10	P09837	*C. dromedarius*	HCLEKNDCSR	79	88	1318.56	2
11	P09837	*C. dromedarius*	HCLEKNDCSRDDQCEGNK	79	96	2264.91	1
12	P09837	*C. dromedarius*	HCLEKNDCSRDDQCEGNKK	79	97	2393.00	1
13	P09837	*C. dromedarius*	KCCFSSCAMR	97	106	1322.51	1
14	P09837	*C. dromedarius*	CCFSSCAMR	98	106	1161.39	1
15	P09837	*C. dromedarius*	CLDPVTEDSFQ	107	117	1310.56	13
16	S9XKL5	*C. ferus*	**SCTVPLMEPVLK**	130	141	1389.71	1

The table is classified by the start aa residues from the N-terminal sequence. Obtained data matches against UniprotKB taxonomy cetartiodactyla (SwissProt + Trembl) database. Molecular masses (M_*r*_) of peptides are expressed in Da. Spectra indicates the number of spectra permitting the identification of peptides. Charge corresponds to the number of charges (z) of multi-charged ions precursors having given the MS/MS spectra. Tryptic peptides connecting the DI and DIIa domains of the protein, including or not the tetrapeptide VSSP, are in bold. Numbering of the *C. ferus* peptide sequence is from KB016488 Genomic DNA Translation

## Discussion

Here we provide the complete camel WAP mRNA sequence (408 nucleotides open reading frame) that encodes a N-terminal signal peptide of 19 aa residues and a mature protein of 117 aa residues, of which the molecular mass is 12,564 Da, and we report the occurrence in *C. bactrianus* from Kazakhstan of a WAP genetic variant, exhibiting a molecular mass of 12,596 Da (unphosphorylated isoform). cDNA sequencing revealed a transition G/A, which modifies an amino acid residue of the mature protein (V12 M), accounting for the mass difference (32 Da) observed between this new genetic variant and the originally described variant [9].

### Camel WAP is phosphorylated

Camel WAP contains five potential phosphorylation sites (S-X-A code) per molecule (S17, S18, S19, S58, and S87), meanwhile rat WAP has only three potential phosphorylation sites [29]. Whereas rat WAP is phosphorylated, it was reported that mouse WAP is apparently non-phosphorylated [8]. From mass data, it is clear that only one site is phosphorylated in camel WAP. Given the extremely constrained and compact structure of the molecule with 8 S-S bridges, essential for folding and functionality of the protein, it is very likely that S58 which is located within the additional sequence connecting the two 4-DSC domains, is the only one seryl residue which is alternatively phosphorylated in camel. Therefore, the other potential phosphorylation sites, namely S17, S18, S19 and S87, should not be phosphorylated. Indeed, WAP contains 16 cysteinyl (C) residues, all of which being involved in disulfide bridges. C residues appear in unique arrangements, divided into two domains. Camel WAP consists of two 4-DSC domains, which are located between aa residues 9 and 55 (DI) and 64 and 111 (DIIA). Each domain begins with a six aa WAP motif (9GQAV**C**P14 and 64KDGR**C**P69), containing the first C residue of the 8 found in the domain.

### The usage of an unlikely intron cryptic splice site is responsible for the insertion of 4 amino acid residues in the major camel WAP isoform

**Fig. 5:**
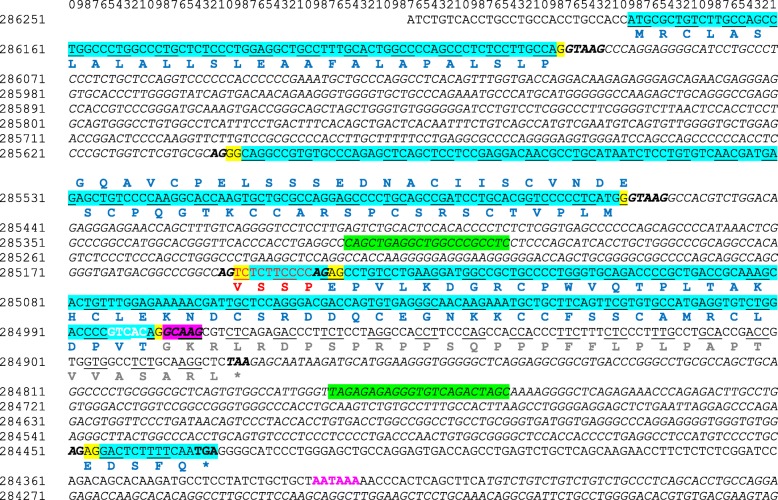
Complete sequence of *C. dromedarius* WAP gene available in GenBank (NCBI, LOC105095719). Primer pairs used for PCR and gDNA sequencing are highlighted in green. Introns and intergenic sequences are in italics. Intron donor and acceptor sites are bolded. The effective intron donor site (GCAAG) is highlighted in fuchsia. The reading frame in WAP gene is preserved through a 2–1 exon phase (nucleotides involved at the 5′- and 3′-ends are highlighted in yellow). Triplet codons encoding aa are given in blue and encoding specific to camel WAP tetrapeptide VSSP in red. The wrong protein sequence is indicated in grey and polyA site (AATAAA) is in purple. Stop codons are indicated with *: the correct one is in blue and “algorithm” predicted is in grey

Camel WAP shows the higher sequence identity at the aa level (76%) to porcine WAP and much lower aa sequence identities to the WAP from dog (65%), rabbit (51%), rat (40%) and mouse (39%). The comparison of camel WAP sequence with that of the other 5 eutherian species in which the WAP gene is expressed *(Sus scrofa, Canis familiaris, Oryctolagus cuniculus, Rattus norvegicus* and *Mus musculus)*, shows a 4 aa residues (56VSSP59) insertion in the camel polypeptide chain at the beginning of the second 4-DSC domain (Fig. 1). From the *Camel dromedarius* gene sequence (GenBank LOC105095719) this appears to be the consequence of the usage of an unlikely intron cryptic splice site extending camel exon 3 on its 5′ side by 12-nucleotides, whereas in the other 5 species the canonic 3′ end of intron 2 is used (Fig. 5). Indeed, there are two potential intron donor splice sites responding to all requirements of splicing recognition signal: *CCCGGCC****AG***│TCTCTTCCCCAG│AGCCTGTCCTG (vertical bars materializing possible intron end recognition sites). Paradoxically it is the weakest site (*CCCGGCC****AG***), with two purine residues (GG) interrupting the polypyrimidine tract, that is preferentially used by the splicing machinery. We confirmed this sequence, by sequencing a 580-bp fragment amplified from genomic DNA encompassing integrally exon 4 and flanking intron sequences (Fig. 5).

Alternative pre-mRNA splicing generates multiple protein isoforms from a single gene. Leading to a shorter or longer peptide chain, a non-allelic exon-skipping event may occur during the course of the pre-mRNA splicing. It is thought to be provoked by the weakness (unperfected complementarities between splice site signals and corresponding small ribonuclear protein particles that make up the spliceosome) in the consensus sequences, either at the 5′ and/or 3′ splice junctions or at the branch point, or both [30]. As far as camel WAP is concerned, even though cDNA sequencing allowed characterizing a single transcript we could not exclude the existence of a non-allelic short isoform of camel WAP, encoded by a shorter mRNA arising from the usage, as in the other species, of the canonic 3′ splice site (TCTCTTCCCC***AG*)**, which is indeed strongly suggested by the results of LC-MS/MS analyses.

### Intron 3 of camel WAP gene is a GC-AG intron type

During the maturation process of pre-mRNA, introns are precisely removed by a large ribonucleoprotein complex: the spliceosome. This splicing step requires splice signals at the 5′ and the 3′ ends of the intron to be removed and a branch point [31]. In their vast majority, introns begin with the standard form dinucleotide GT at the 5′ splice site and terminate with the dinucleotide AG at the 3′ splice site, so-called GT-AG introns. This rule hold in most cases, however some exceptions have been found [32]. For example, at the 5′ terminus of a few introns, a dinucleotide GC can be occasionally found [33]. Based on the data sets derived from annotated gene structures, it has been reported that GC donor sites account for less than 1% of annotated donor sites and possess a strong consensus sequence [33]. GC-AG introns are processed by the same splicing pathway (U2-type spliceosome) as conventional GT-AG introns [34]. GC-AG introns works in balance with alternative GT splice donor and uses alternative donor and acceptor splice sites, and lack a reasonable poly pyrimidine tract [33]. In humans, about 0.7% of GC-AG introns are involved in regulated splicing [35]. In *Caenorhabditis elegans,* experiments indicate that the conserved C at the + 2 position of the tenth intron of the let-2 gene is essential for developmentally regulated alternative splicing [35]. In camel WAP gene, the C might allow the splice donor to function as a very weak splice site that works in balance with an alternative GT splice donor. In this respect, the only possibility would be the use of the GTCAC site, 7 nt upstream of the GCGAG. Such an assumption would modify the 3′ acceptor splice site of intron 3 to maintain a frame of reading in phase and to cause the loss of 3 aa residues: V and T (5′ side of intron 3) and E (3′ side of intron 3) in the camel WAP sequence. The C-terminal sequence of the protein described by Beg and co-workers [9] terminates with the peptide sequence DP**VTE**DSFQ. The protein sequence deduced from our cDNA sequencing, in accordance with the molecular mass determined from LC-MS, terminates with the identical DPVTEDSFQ peptide sequence. The usage of the postulated alternative GT donor splice site cannot be excluded. However, then to preserve the reading frame in phase, the upstream intron should end with the second AG (284,453/4) highlighted in yellow at Fig. 5. However, we were unable to detect a DPDSFQ C-terminal, as well in LC-MS/MS as through cDNA sequencing. Surprisingly, in WAP gene, available in GenBank (NCBI, LOC105095719) exon 3 is prolonged with 99 nucleotides encoding 33 aa residues until the occurrence of a potential TAA stop codon, which would make exon 4 ineffective. From our results, in agreement with the protein structure first reported [9] and our results, the use of this GC donor site is more than likely. Especially since it was reported that alternative GC-AG introns show a compensatory effect in terms of a dramatic increase in consensus at the donor (AG-GCAAG) as well as at the polyYx(C/T)AG-G acceptor exon positions [33].

## Conclusions

In this study, combining proven proteomic and molecular biology approaches, three main findings in respect to camel WAP are provided. The first one is the identification of a new genetic variant (B), originating from a transition G = > A, leading to a codon change (GTG/ATG) in the nucleotide sequence of a Bactrian cDNA, which modifies a single amino acid residue at position 12 of the mature protein (V12 M). The second is the detection of two transcripts coding for camel WAP, of which the major one is arising from an improper and unusual processing of a unique pre-mRNA, due to a cryptic splice site usage. This phenomenon leads to the gain or loss of 4 amino acid residues (56VS**S**P59), of which one serine residue (in bold and underlined) is alternatively phosphorylated. Such a genetic polymorphism and splicing events generate a molecular sequence diversity that might account for physiochemical properties of camel WAP that would be quite different, and might contribute unique properties to camel milk. Finally, we report here the occurrence of a GC-AG intron-type (intron 3) in camel gene encoding WAP, showing a compensatory effect in terms of a dramatic increase in consensus at the acceptor exon position.

## Abbreviations

4-DSC: Four-disulfide cores
aa: Amino acid
bp: Base pair
LC-ESI-MS: Liquid chromatography-electrospray ionization-tandem mass spectrometry
LC-MS/MS: Liquid chromatography coupled to tandem mass spectrometry
UTR: Untranslated transcribed region
WAP: Whey acidic protein
